# Photo-Fenton Degradation of AO7 and Photocatalytic Reduction of Cr(VI) over CQD-Decorated BiFeO_3_ Nanoparticles Under Visible and NIR Light Irradiation

**DOI:** 10.1186/s11671-019-3206-5

**Published:** 2019-12-30

**Authors:** Tao Xian, Lijing Di, Xiaofeng Sun, Hongqin Li, Yongjie Zhou, Hua Yang

**Affiliations:** 1 College of Physics and Electronic Information Engineering, Qinghai Normal University, Xining, 810008 China; 2 State Key Laboratory of Advanced Processing and Recycling of Non-Ferrous Metals, Lanzhou University of Technology, Lanzhou, 730050 China

**Keywords:** BiFeO_3_ nanoparticles, Carbon quantum dots, CQD/BiFeO_3_ composites, Photocatalysis

## Abstract

In this work, the carbon quantum dot (CQD)–decorated BiFeO_3_ nanoparticle photocatalysts were prepared by a hydrothermal method. The TEM observation and XPS characterization indicate that the CQDs are well anchored on the surface of BiFeO_3_ nanoparticles. Acid orange 7 (AO7) and hexavalent chromium (Cr(VI)) were chosen as the model pollutants to investigate the photocatalytic/photo-Fenton degradation and photocatalytic reduction performances of the as-prepared CQD/BiFeO_3_ composites under visible and near-infrared (NIR) light irradiation. Compared with bare BiFeO_3_ nanoparticles, the CQD/BiFeO_3_ composites exhibit significantly improved photocatalytic and photo-Fenton catalytic activities. Moreover, the composites possess good catalytic stability. The efficient photogenerated charges separation in the composites was demonstrated by the photocurrent response and electrochemical impedance spectroscopy (EIS) measurements. The main active species involved in the catalytic degradation reaction were clarified by radicals trapping and detection experiments. The underlying photocatalytic and photo-Fenton mechanisms are systematically investigated and discussed.

## Background

In recent decades, wastewater containing heavy metal ions and organic compounds brings serious damages for environment and human beings. As one of common heavy metal ions, hexavalent chromium (Cr(VI)) derived from electroplating, leather tanning, and printing poses a serious threat for our health owing to its high toxicity [1]. On the other hand, most of organic pollutants (such as dyes) are also toxic and non-biodegradable, which destroy our living environment [2]. Up to now, many techniques have been developed to eliminate organic pollutants and reduce Cr(VI) to Cr(III) [3–5]. Among these methods, photocatalytic and photo-Fenton-like catalytic techniques are regarded to be the promising methods for efficient degradation of organic contaminants and Cr(VI) reduction in wastewater because of their inexpensive cost, non-selectivity, and simplicity of operation [6–9]. The basic steps involved in a photocatalytic degradation process can be described as follows: excitation of photocatalysts, separation and migration of the photogenerated charges, generation of active species on the surface of catalysts, and decomposition of organic compound as well as reduction of Cr(VI) caused by the redox reaction of active species and photo-induced charges [10, 11]. The photo-Fenton-like catalytic reaction is based on the synergistic effects of the Fenton reaction and photocatalytic process. The generation of active species during the Fenton reaction process can be promoted after the introduction of suitable light irradiation, which leads to improved catalytic activity [12, 13]. However, the wide application of photocatalytic and photo-Fenton-like catalytic techniques is limited due to the large bandgap of photocatalysts only responding to UV light (which accounts for ~ 5% of sunlight energy) and their low charge separation efficiency [14]. Generally, it is known that the visible light and near-infrared (NIR) light occupy ~ 45% and ~ 46% of solar energy, respectively, and their application has received a great deal of interest [15, 16]. As a result, the development of broad spectrum (UV-vis-NIR) active catalysts with efficient separation of photogenerated charges is very important for their practical applications [17–20]. Up to now, the iron-contained catalysts with narrow bandgap are considered as ideal candidates in the photocatalytic and photo-Fenton-like catalytic applications [21–25].

As one of typical iron-contained catalysts, BiFeO_3_ with perovskite-type structure is known to be an interesting visible light-driven photocatalytic and photo-Fenton-like catalytic material for the degradation of dyes [26–34]. Nevertheless, its catalytic activity is not so strong to meet the application requirements owing to the high recombination rate of photogenerated charges. Moreover, the light response range of BiFeO_3_ needs to be further extended to NIR light region for effective utilization of sunlight energy. Therefore, many strategies have been used to overcome these shortcomings [35–40].

Carbon quantum dots (CQDs), as an important class of zero-dimensional nanocarbon material, have attracted considerable attentions due to its distinct properties, such as large surface area, low toxicity, high biocompatibility, good water solubility, high chemical stability, good electrical conductivity, and excellent optical properties [41–44]. These prominent properties make it a promising candidate for the practical application in different fields [41–44]. More importantly, the photoexcited CQDs are demonstrated to be an excellent electron donors and acceptors to promote the separation of photogenerated charges in photocatalysts [45]. On the other hand, CQDs are found to be an unique up-converted photoluminescence material, which allows the generation of short-wavelength emission light (from 450 to 750 nm) by the excitation of long-wavelength light (NIR light, from 700 to 1000 nm) [42, 44]. The up-converted emission light can be employed as the excitation light for the production of photogenerated charges in the semiconductors, which extends their light response region [45]. As a result, incorporation of CQDs with photocatalysts is demonstrated to be a promising way to form excellent hybrid composite photocatalysts [46–52]. Chen et al. prepared CQD/BiFeO_3_ nanocomposites and found their enhanced visible light photocatalytic activity for the dye degradation [53]. To the best of our knowledge, however, there is no work devoted to the photo-Fenton dye degradation and photocatalytic Cr(VI) reduction performances of CQD/BiFeO_3_ composite photocatalysts under visible or NIR light irradiation.

In this work, the CQD/BiFeO_3_ composite photocatalysts were prepared by a hydrothermal route. Their photocatalytic and photo-Fenton-like catalytic performance for acid orange 7 (AO7) degradation as well as photocatalytic Cr(VI) reduction activity under visible and NIR light irradiation were systematically investigated. The corresponding catalytic mechanism was proposed.

## Methods

### Preparation of CQDs

The CQDs were prepared by a hydrothermal method [54]. Glucose (1 g) was added into distilled water (80 ml) under magnetic stirring and ultrasonic treatment to obtain a homogeneous solution. Subsequently, this solution was transferred into a 100-mL Teflon-lined stainless steel autoclave and heated at180 °C for 4 h. After the reaction, the resultant solution was filtered by filter paper twice, and then, the reddish-brown CQDs suspension was obtained.

### Fabrication of CQD/BiFeO_3_ Composites

**Fig. 1:**
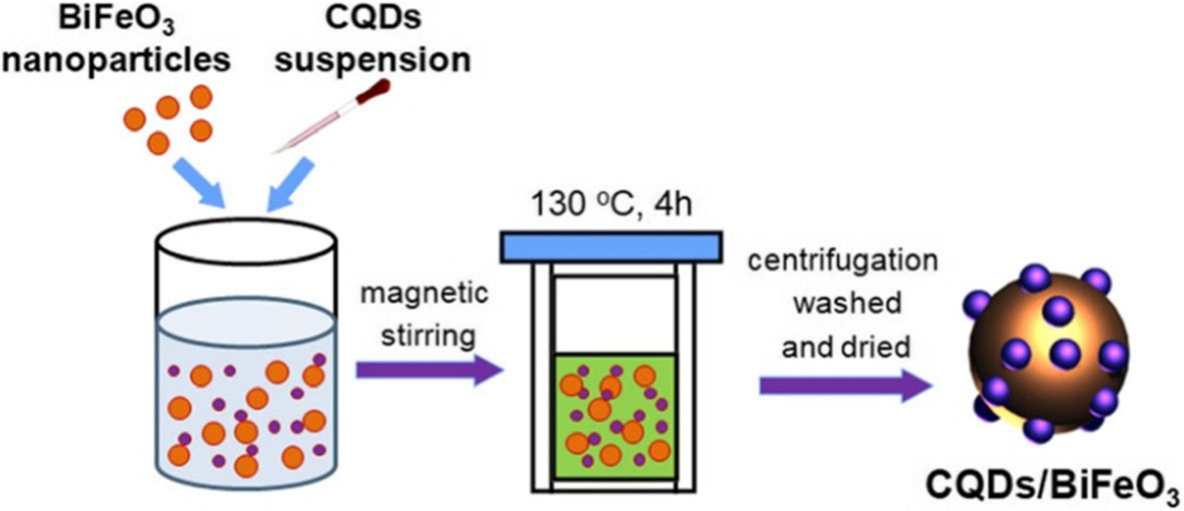
The schematic illustration of preparation process for CQDs/BiFeO_3_ composite

BiFeO_3_ nanoparticles were prepared through a polyacrylamide gel route as reported in the literature [55]. The CQD/BiFeO_3_ composites were fabricated as follows (Fig. 1): BiFeO_3_ nanoparticles (0.1 g) were introduced into distilled water (70 ml), followed by ultrasonic treatment for 0.5 h to obtain uniform suspension. After that, a certain amount of CQD suspension was added drop by drop into the BiFeO_3_ suspension under magnetic stirring. The mixture was moved into the Teflon-lined stainless steel autoclave (100 ml) and heated at130 °C for 4 h. Finally, the product was collected by centrifugation, washed with deionized water, and dried at 60 °C for 8 h. To explore the impact of the CQDs content on the catalytic actives of the composites, a series of CQD/BiFeO_3_ composites with different mass contents of CQDs were prepared by adding different volumes of CQDs suspension (3, 6, 12, and 24 ml). These composites were correspondingly named as 3C/BFO, 6C/BFO, 12C/BFO, and 24C/BFO.

### Photo-Fenton Catalytic and Photocatalytic Degradation of Dye

The photo-Fenton catalytic performance of the as-prepared CQD/BiFeO_3_ composites was investigated toward the degradation of AO7 separately irradiated by visible light (300-W xenon lamp with a 420-nm cutoff filter) and NIR light (300-W xenon lamp with a 800-nm cutoff filter). In a typical experiment, the photocatalyst (0.1 g) was placed into AO7 solution (200 ml, 5 mg/L), and magnetically stirred in dark for 0.5 h to achieve a adsorption-desorption equilibrium between the photocatalyst and AO7 molecules. Subsequently, a certain amount of H_2_O_2_ solution was added into the suspension, and the xenon lamp was turned on to start the catalytic reaction. In the catalytic process, a small amount of the reaction solution (2 ml) was taken and centrifuged to eliminate the catalyst. The absorbance of the supernatant was measured by a UV-vis spectrophotometer at 484 nm to obtain the AO7 concentration. On the other hand, the photocatalytic degradation of AO7 over the samples was performed to evaluate their photocatalytic activities under the same conditions in the absence of H_2_O_2_.

The recycling catalytic experiments were carried out to test the catalytic reusability of the samples. After the first catalytic experiment, the catalyst was separated from the solution by centrifugation, washed with deionized water, and dried. The collected catalyst was added into the new dye solution for the next catalytic reaction with the same condition.

To confirm the reactive species involved in the photocatalytic and photo-Fenton catalytic degradation processes, the active species trapping experiments were performed by adding several scavengers under the same conditions as mentioned above. Ethanol (10% by volume) and ammonium oxalate (AO, 2 mM) were used as the scavengers of hydroxyl (·OH) and photogenerated holes (h^+^), respectively [56]. N_2_ purging can expel the dissolved O_2_ in the solution, leading to the inhibition of superoxide (·O_2_^−^) generation.

### Photocatalytic Reduction of Cr(VI)

Cr(VI) was employed as another model pollutants to measure photocatalytic activity of the samples. The photocatalytic reduction process of Cr(VI) to Cr(III) was similar to that of the dye degradation. The initial concentration of Cr(VI) was 10 mg/l and the photocatalyst dosage was 0.2 g in 200 ml Cr(VI) solution (i.e., 1 g/l). The initial pH value of the Cr(VI) solution was adjusted by H_2_SO_4_ to 2~3. The residual concentration of Cr(VI) solution was detected by UV-vis spectrophotometer using the diphenylcarbazide (DPC) method [57].

### Hydroxyl Radical Detections

Fluorimetry was employed to detect the ·OH radicals generated on the irradiated samples by using terephthalic acid (TA) as a probe molecule. Generally, the ·OH will react with TA to generate highly fluorescent compound, 2-hydroxyterephthalic acid (TAOH). The information of ·OH can be detected through measuring the photoluminescence (PL) intensity of TAOH with the excitation wavelength of ~ 315 nm. Typically, the TA was introduced into NaOH solution (1.0 mmol l^−1^) to obtain TA solution (0.25 mmol l^−1^). The catalyst (60 mg) was placed into TA solution (100 ml) under magnetically stirring for several minutes. After that, a certain amount of H_2_O_2_ was dissolved into above mixture, which was irradiated by visible light (300-W xenon lamp with a 420-nm cutoff filter) or NIR light (300-W xenon lamp with a 800-nm cutoff filter). At given intervals of irradiation, 3 ml of the reaction solution was sampled and centrifuged to remove the catalyst. The PL spectra of the supernatant were determined by fluorescence spectrophotometer. On the other hand, the generation of ·OH in the photocatalytic reaction was also measured under the same conditions without the addition of H_2_O_2_.

### Characterization

The phase purity of the samples was examined by X-ray powder diffraction (XRD) and Fourier-transform infrared spectroscopy (FTIR). The morphology and microstructure of the samples were observed by field-emission transmission electron microscopy (TEM). The chemical states of the surface elements on the samples were detected by X-ray photoelectron spectroscopy (XPS). The ultraviolet-visible (UV-vis) diffuse reflectance spectra of the samples were recorded through a TU-1901 double beam UV-vis spectrophotometer. The PL spectra of the samples were determined by a fluorescence spectrophotometer. The transient photocurrent response and electrochemical impedance spectroscopy (EIS) measurements were carried out on an electrochemical workstation with a three-electrode system. The working electrode fabrication and test procedures were similar to those previously reported [56]. Particularly, the photocurrent response measurement was performed under visible light (300-W xenon lamp with a 420-nm cutoff filter) irradiation.

## Results and Discussion

### XRD Analysis

**Fig. 2:**
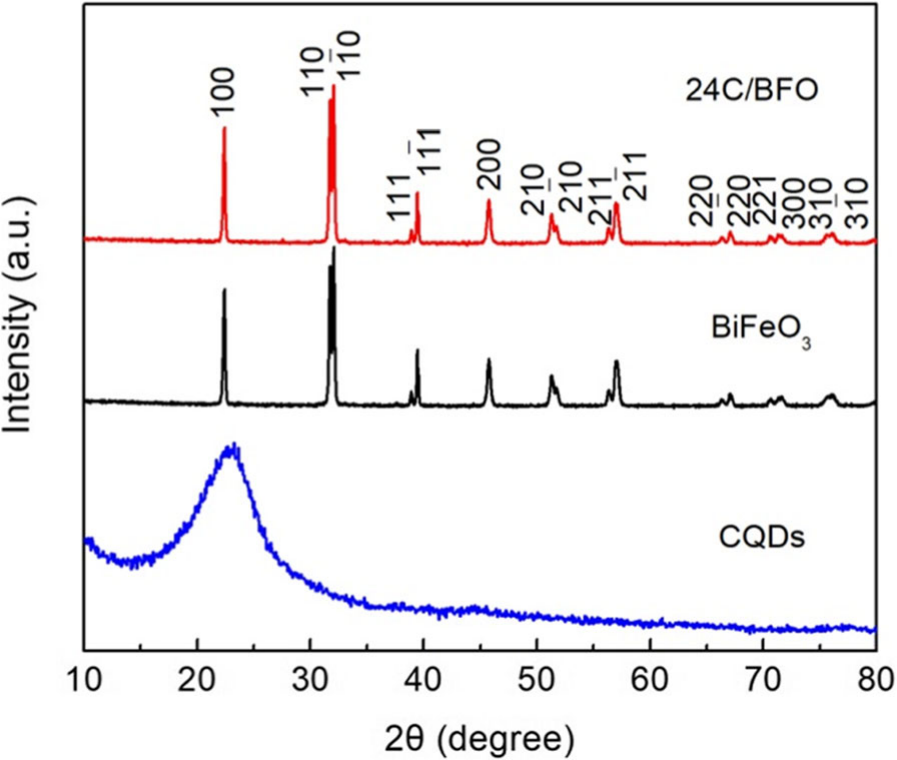
XRD patterns of BiFeO_3_, CQD, and the 24C/BFO composites

Figure 2 presents the XRD patterns of BiFeO_3_, CQDs, and 24C/BFO. The BiFeO_3_ and 24C/BFO sample show similar diffraction patterns, which can be readily indexed to the rhombohedral BiFeO_3_ phase (JCPD file no: 74-2016). No trace of impurities, such as Fe_2_O_3_ and Bi_2_O_3,_ is found. The results indicate that the high-purity BiFeO_3_ is obtained and the introduction of CQDs and hydrothermal treatment do not obviously change the crystal structure of BiFeO_3_. From the XRD pattern of CQDs, one can see that a broad diffraction peak is observed at ~ 23.5°, which is mainly attributed to the amorphous structure of CQDs. Notably, for the composite, no characteristic diffraction peaks of CQDs are detected owing to the low content of CQDs in the 24C/BFO sample. To confirm the existence of CQDs in the composite, the FTIR characterization is performed.

### FTIR Analysis

**Fig. 3:**
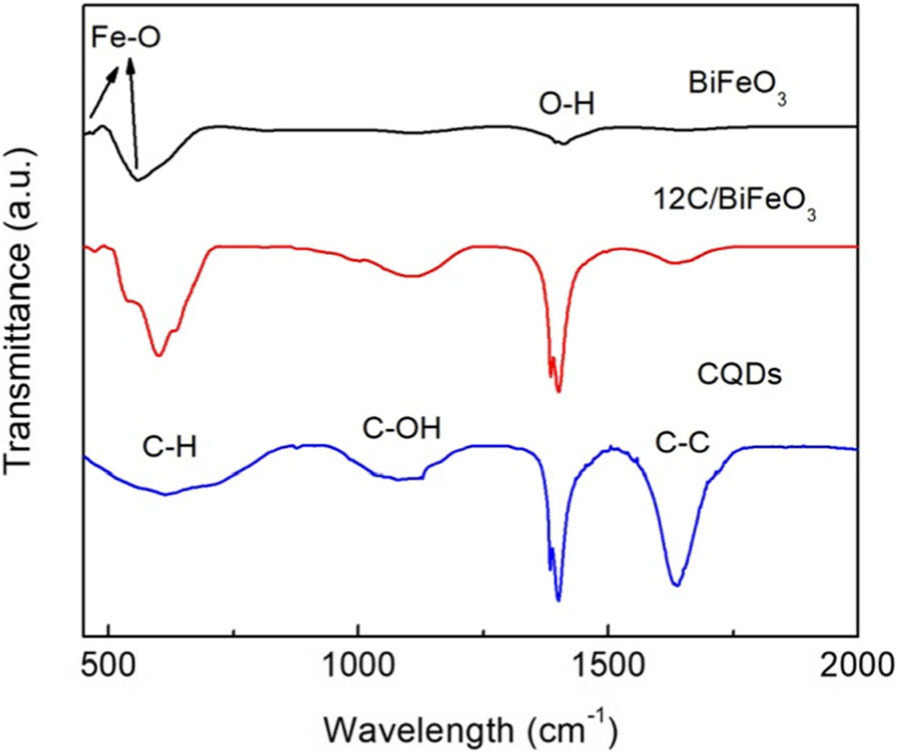
FTIR spectra of BiFeO_3_, CQD, and the 12C/BFO composites

Figure 3 shows the FTIR spectra of BiFeO_3,_ CQD, and 12C/BFO composites. In the case of bare BiFeO_3_, the peaks at ~ 440 cm^−1^ and ~ 560 cm^−1^ are assigned to the stretching and bending vibrations of Fe–O, which is consistent with the reported result [55]. For the CQDs, the deformation vibration for C–H at ~ 638 cm^−1^, the stretching vibration for C–C at ~ 1630 cm^−1^, and C–OH stretching at ~ 1120 cm^−1^ are found [58]. In addition, the characteristic peaks of BiFeO_3_ and CQDs are detected in the spectrum of 12C/BFO composite. The results suggest the existence of CQDs and BiFeO_3_ in the composite. Moreover, the peak located at ~ 1380 cm^−1^ is attributed to the stretching vibration of O–H from the absorbed H_2_O [59].

### Optical Absorption Property

**Fig. 4:**
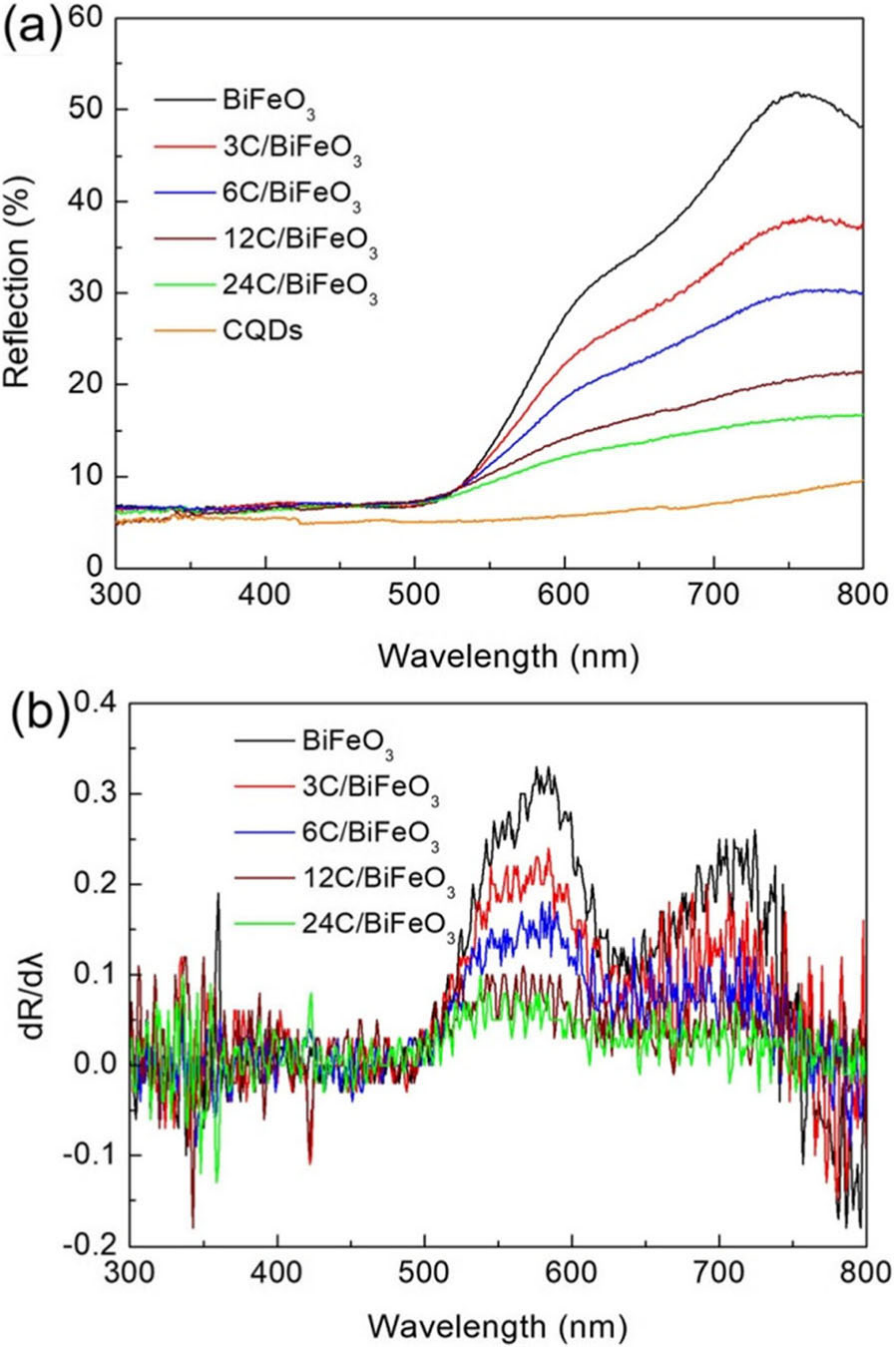
**a** UV-vis diffuse reflectance spectra of BiFeO_3_, CQD, and CQDs/BiFeO_3_ composites. **b** The corresponding first derivative of the diffuse reflectance spectra

It is well established that the optical absorption property of nanomaterials has an important effect on their performance [60, 61]. The optical absorption property of BiFeO_3_, CQD, and CQDs/BiFeO_3_ composites were investigated by UV-vis diffuse reflectance spectra, as shown in Fig. 4a. Compared with BiFeO_3_, the CQD/BiFeO_3_ composites exhibit obviously enhanced optical absorption capability in the entire UV-vis light region. It is worth noting that the optical absorption intensity of the composites gradually increases with increasing the content of CQDs. This phenomenon can be attributed to the strong light absorption of CQDs in the UV-vis light region. To obtain the light absorption edge of the samples, the first derivative curves of the UV-vis diffuse reflectance spectra are carried out (Fig. 4b), in which the peak wavelength is considered to be the absorption edge of the samples [62]. It is found that absorption edges of BiFeO_3_ and CQD/BiFeO_3_ composites are located at ~ 588 nm, suggesting that the decoration of CQDs does not change the bandgap energy of BiFeO_3_.

### XPS Analysis

**Fig. 5:**
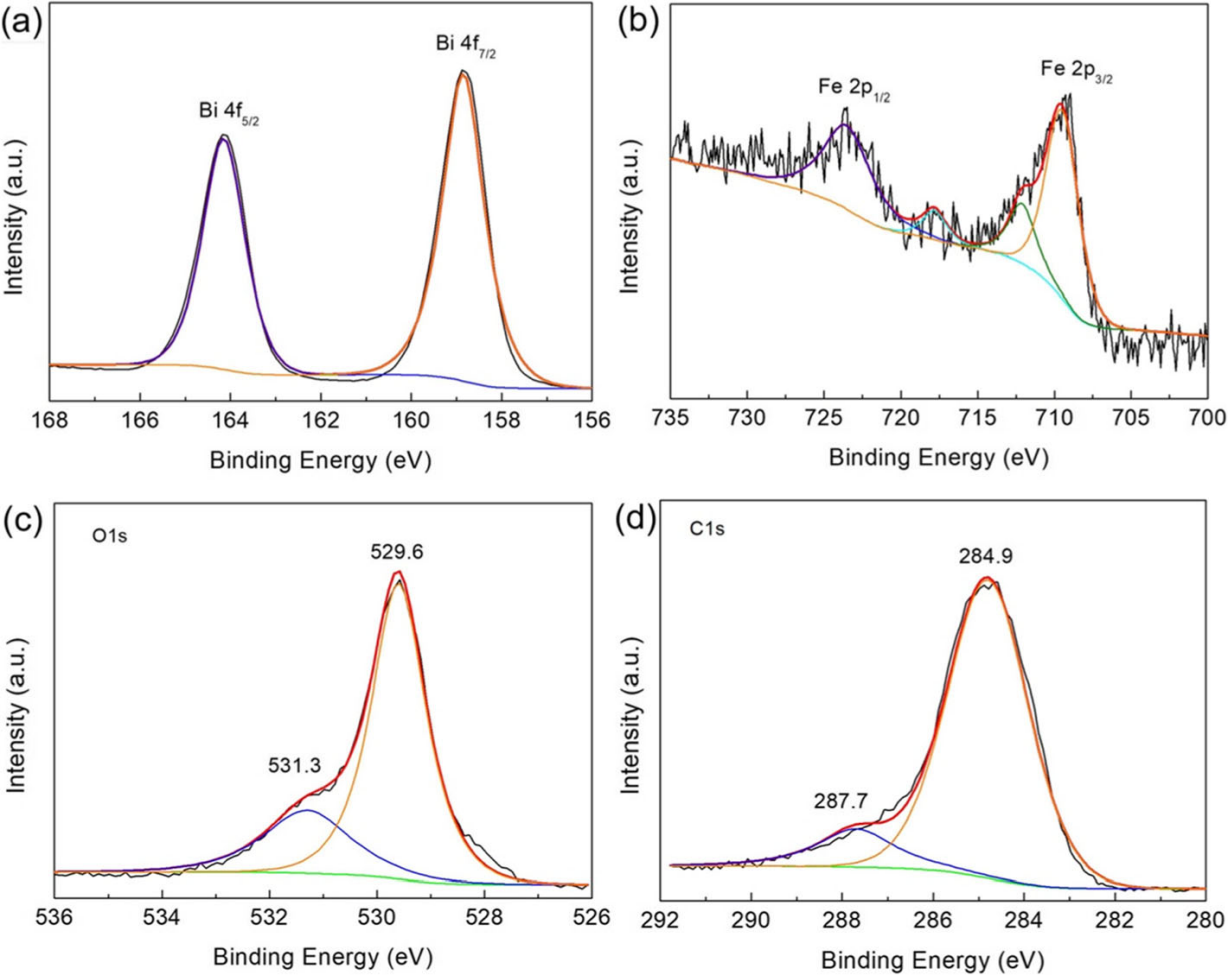
High-resolution XPS spectra of **a** Bi 4f, **b** Fe 2p, **c** O 1s, and **d** C 1s for the 12C/BFO composite

The chemical states of elements in the 12C/BFO sample were monitored by XPS and the results are presented in Fig. 5. On the Bi 4f XPS spectrum (Fig. 5a), the observed two strong peaks at 164.1 (Bi 4f_5/2_) and 158.8 eV (Bi 4f_7/2_) demonstrate the existence of Bi^3+^ in the composite [63]. In Fig. 5b, the Fe 2p XPS spectrum indicates two obvious peaks at 723.6 and 709.6 eV, which are attributed to Fe 2p_1/2_ and Fe 2p_3/2_. Notably, the broad peak of Fe 2p_3/2_ can be divided into two peaks at 712.0 and 709.6 eV, corresponding to Fe^3+^ and Fe^2+^, respectively [40]. In addition, it is seen that the satellite peak of Fe 2p_3/2_ is found at 717.8 eV. As shown in the XPS spectrum of O 1s (Fig. 5c), the obvious peak located at 529.6 eV is attributed to the lattice oxygen and the shoulder peak at 531.3 eV belongs to the chemisorbed oxygen of surface vacancies [64]. For the XPS spectrum of C 1s (Fig. 5d), the signal of C 1s can be divided into two distinct peaks. The major peak at ~ 284.9 eV is ascribed to the C–C bond with sp^2^ orbital, whereas the peak at 287.7 eV is caused by the oxygenated carbon. The results further demonstrate the coexistence of CQDs and BiFeO_3_ in the composite [65].

### Morphology Observation

**Fig. 6:**
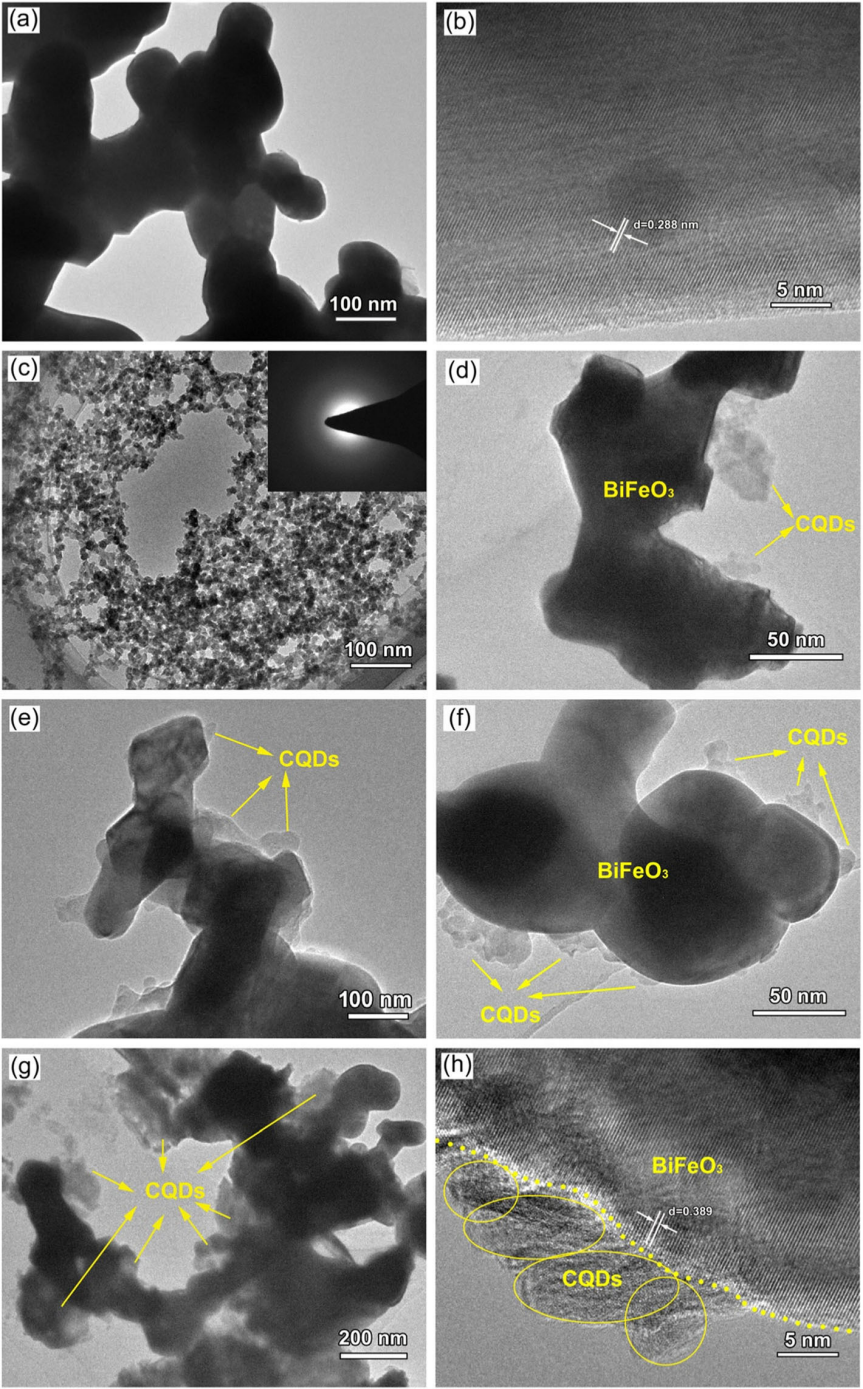
**a** and **b** TEM and HRTEM images of bare BiFeO_3_ nanoparticles, respectively; **c** TEM image of CQDs; **d**–**g** TEM images of 3C/BFO, 6C/BFO 12C/BFO, and 24C/BFO respectively; **h** HRTEM image of 12C/BFO

The TEM and high-resolution TEM (HRTEM) images of BiFeO_3_ nanoparticles are shown in Fig. 6a and b, respectively. It is seen that the bare BiFeO_3_ possesses a sphere-like shape and smooth surface with an average diameter of ~ 120 nm. The lattice spacing of 0.288 nm belongs to the (110) spacing of BiFeO_3_. The TEM image in Fig. 6c indicates that the CQDs are composed of spherical-like particles with an average particle size of ~ 15 nm. From the TEM image of the CQD/BiFeO_3_ composites (Fig. 6d–g), one can see that the CQDs are decorated on the surface of BiFeO_3_ nanoparticles. The HRTEM image of the 12C/BiFeO_3_ sample (Fig. 6h) reveals the interplanar distance of 0.389 nm corresponding to the (012) plane of BiFeO_3_. Alongside of BiFeO_3_, the decorated CQDs exhibit amorphous characteristic. This result suggests the formation of hybrid composite structure between BiFeO_3_ and CQDs.

**Fig. 7:**
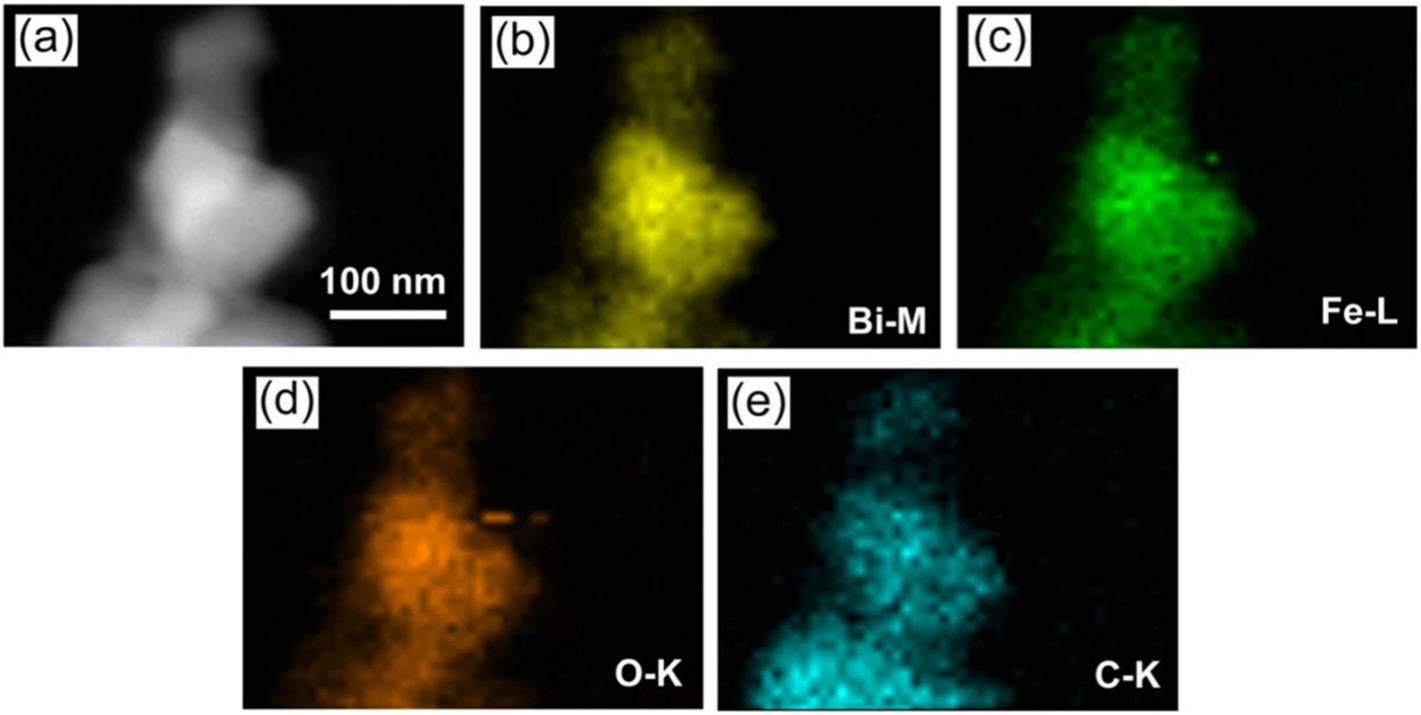
**a** DF-STEM image of the 12C/BFO composite. **b**–**e** The corresponding energy dispersive X-ray elemental mapping images

The dark-field scanning TEM (DF-STEM) image and the corresponding elemental mappings of the 12C/BFO sample are shown in Fig. 7a–e, respectively. The results reveal that the sample presents not only uniform distribution of the Bi/Fe/O elements but also uniform distribution of the C element. This confirms that CQDs are uniformly assembled on the surface of BiFeO_3_ nanoparticles.

### Photo-Fenton Catalytic and Photocatalytic Performance

**Fig. 8:**
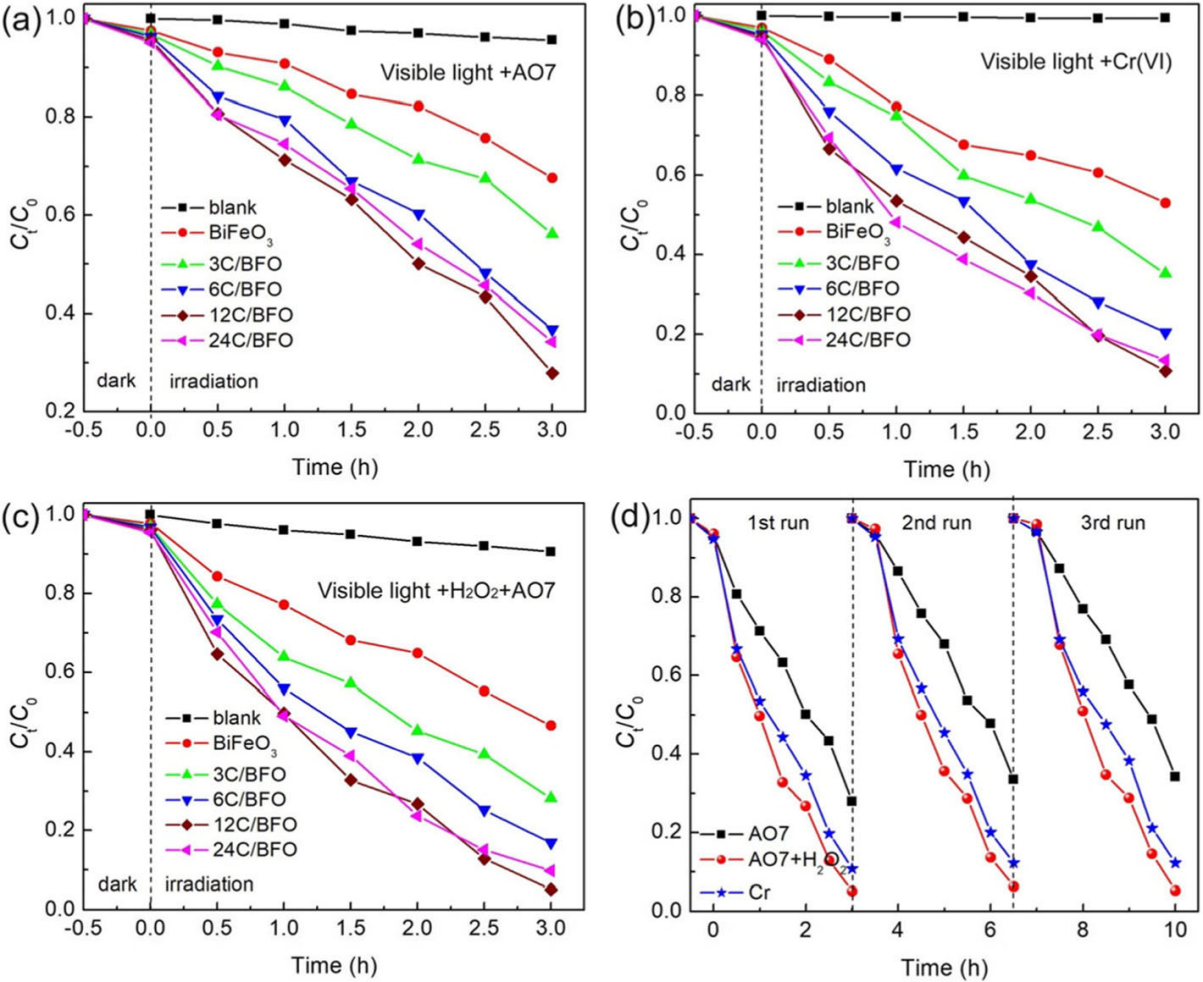
**a** Photocatalytic degradation of AO7, **b** photocatalytic reduction of Cr(VI), and **c** photo-Fenton catalytic degradation of AO7 over BiFeO_3_ and CQD/BiFeO_3_ composites under visible light irradiation. **d** Recyclability of the 12C/BFO composite for photocatalytic degradation of AO7, photocatalytic reduction of Cr(VI), and photo-Fenton catalytic degradation of AO7 under visible light irradiation

The photocatalytic performance of the samples was first assessed by the degradation of AO7 under visible light irradiation, and the result is shown in Fig. 8a. Prior to the photocatalytic reaction, the adsorption (in the dark) and blank (without catalyst) experiments were carried out. A small amount of AO7 (~ 5%) is degraded after 3-h irradiation without catalyst, indicating that the self-degradation of the dye can be neglected. In the photocatalytic reaction, the photodegradation ability of pure BiFeO_3_ is weak and only ~ 33% of AO7 is observed to be decomposed after 3-h exposure. When BiFeO_3_ nanoparticles are decorated by CQDs, the CQD/BiFeO_3_ composites exhibit obviously enhanced photocatalytic activity. Moreover, it is found that the catalytic activities of the composites are highly related to the content of CQDs. Among these composites, the 12C/BiFeO_3_ composite displays the optimal degradation percentage of ~ 73% after 3-h irradiation, which is 2.2 times higher than that of bare BiFeO_3_. However, with further increase of the CQD content (e.g., 24C/BFO), excessive CQDs decorated on the surface of BiFeO_3_ nanoparticles may shield BiFeO_3_ from absorbing visible light, which leads to the decrease of the photocatalytic activity.

In this work, the photocatalytic ability of the samples for the reduction of Cr(VI) under visible light irradiation was also studied, as shown in Fig. 8b. The blank experiment indicates that the reduction of Cr(VI) after 3-h illumination in the absence of catalysts is negligible. It is seen that the CQD/BiFeO_3_ composites possess much higher photocatalytic reduction ability than pure BiFeO_3_. The reduction efficiency of Cr(VI) over the samples increases in the order: BiFeO_3_ < 3C/BFO < 6C/BFO < 24C/BFO < 12C/BFO. The result demonstrates the visible light-driven photocatalytic reduction property of BiFeO_3,_ which can be obviously improved by the decoration of CQDs.

Besides the photocatalytic activity, it is demonstrated that BiFeO_3_ also displays promising photo-Fenton-like catalysis ability. Figure 8c shows the photo-Fenton degradation of AO7 over the samples under visible light irradiation with the addition of H_2_O_2_, from which one can see that the degradation percentage of AO7 in the photo-Fenton-like catalytic process is much higher than that in bare photocatalytic reaction. For example, about 96% of AO7 is photo-Fenton catalytically degraded over 12C/BFO sample under 3-h irradiation, which has a ~ 23% enhancement compared with the photocatalytic degradation of AO7 (~ 73%). In addition, it is found that the photo-Fenton catalytic activities between the samples have an order same to the photocatalytic activities between the samples. This suggests that the CQD/BiFeO_3_ composites can be used as effective photo-Fenton catalysts for the degradation of dyes.

Generally, the reusability of catalysts is regarded as an important parameter for their practical application. According to above catalytic results, the 12C/BFO sample was chosen as the catalyst for the investigation of photocatalytic and photo-Fenton catalytic stabilities. Figure 8d presents the catalytic activities of the 12C/BFO sample during three successive visible light-driven photocatalytic and photo-Fenton catalytic processes. After three consecutive cycles, the catalytic activities of the 12C/BFO sample do not undergo obvious decrease. This indicates that the CQD/BiFeO_3_ composite exhibits good catalytic reusability under visible light irradiation.

**Fig. 9:**
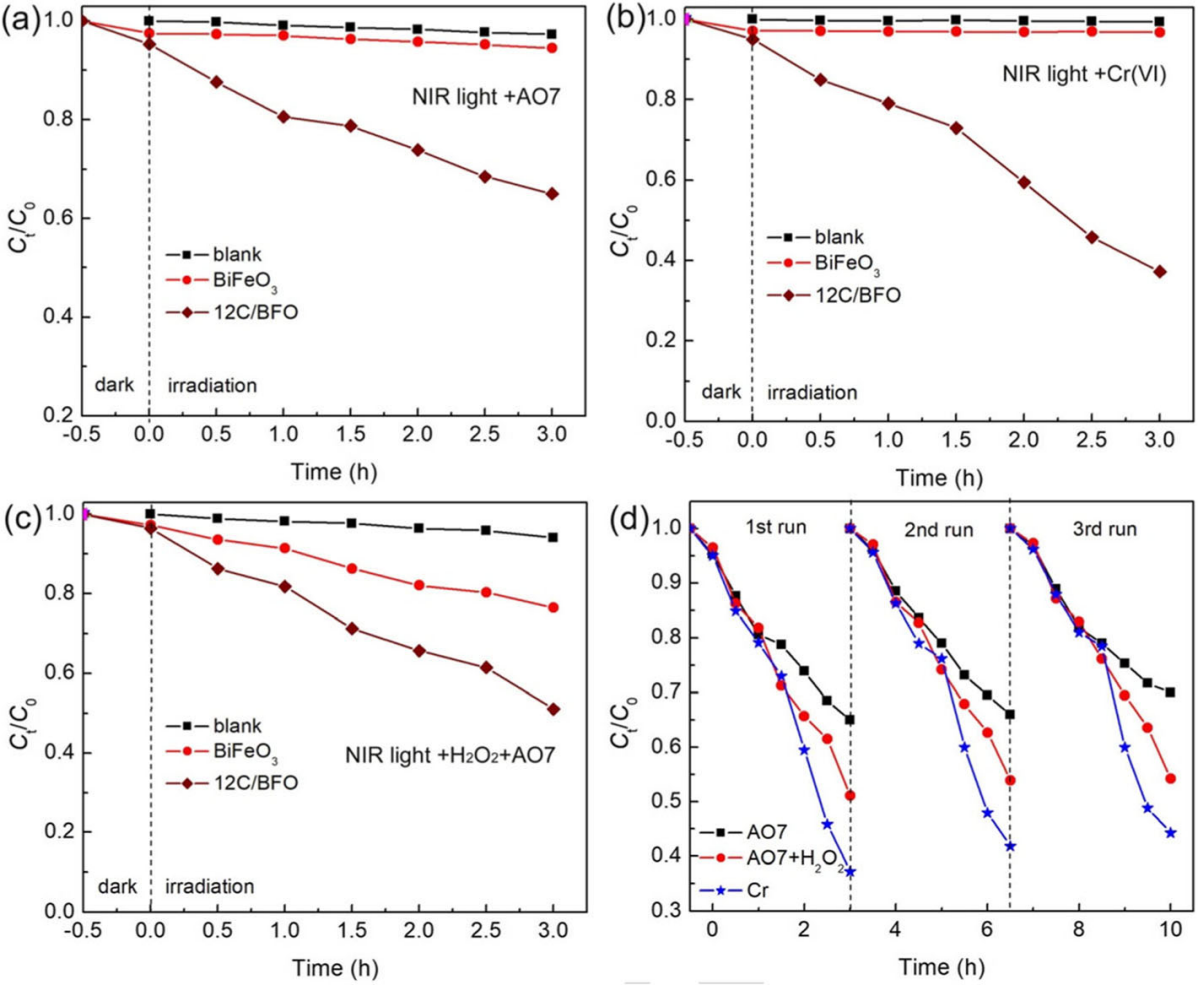
**a** Photocatalytic degradation of AO7, **b** photocatalytic reduction of Cr(VI), and **c** photo-Fenton catalytic degradation of AO7 over BiFeO_3_ and CQD/BiFeO_3_ composites under NIR light irradiation. **d** Recyclability of the 12C/BFO composite for photocatalytic degradation of AO7, photocatalytic reduction of Cr(VI), and photo-Fenton catalytic degradation of AO7 under NIR light irradiation

In this work, the NIR light-driven photocatalytic and photo-Fenton catalytic activities of BiFeO_3_ and 12C/BFO were investigated. Figure 9a–c display the time-dependent photocatalytic degradation of AO7, photocatalytic reduction of Cr(VI), and photo-Fenton catalytic degradation of AO7 over BiFeO_3_ and 12C/BiFeO_3_ under NIR light irradiation, respectively. It can be seen that bare BiFeO_3_ exhibits almost no NIR light photocatalytic activity because it cannot respond to NIR light, while about 22% of AO7 is degraded by BiFeO_3_ during the photo-Fenton catalytic reaction. In contrast, the 12C/BFO sample displays obvious NIR light-driven catalytic activities. After 3-h NIR light irradiation, the photocatalytic degradation of AO7, photocatalytic reduction of Cr(VI), and photo-Fenton degradation of AO7 over the 12C/BFO sample reach ~ 35%, ~ 63%, and ~ 49%, respectively. The result indicates that the introduction of CQDs onto the surface of BiFeO_3_ plays an important role in the enhancement of its NIR light-driven catalytic activity. The NIR light catalytic stabilities of the 12C/BFO sample were also studied by recycling catalytic experiments, as shown in Fig. 9d. It is found that the CQD/BiFeO_3_ composite also has steady NIR light-driven catalytic activity.

### Active Species Trapping

**Fig. 10:**
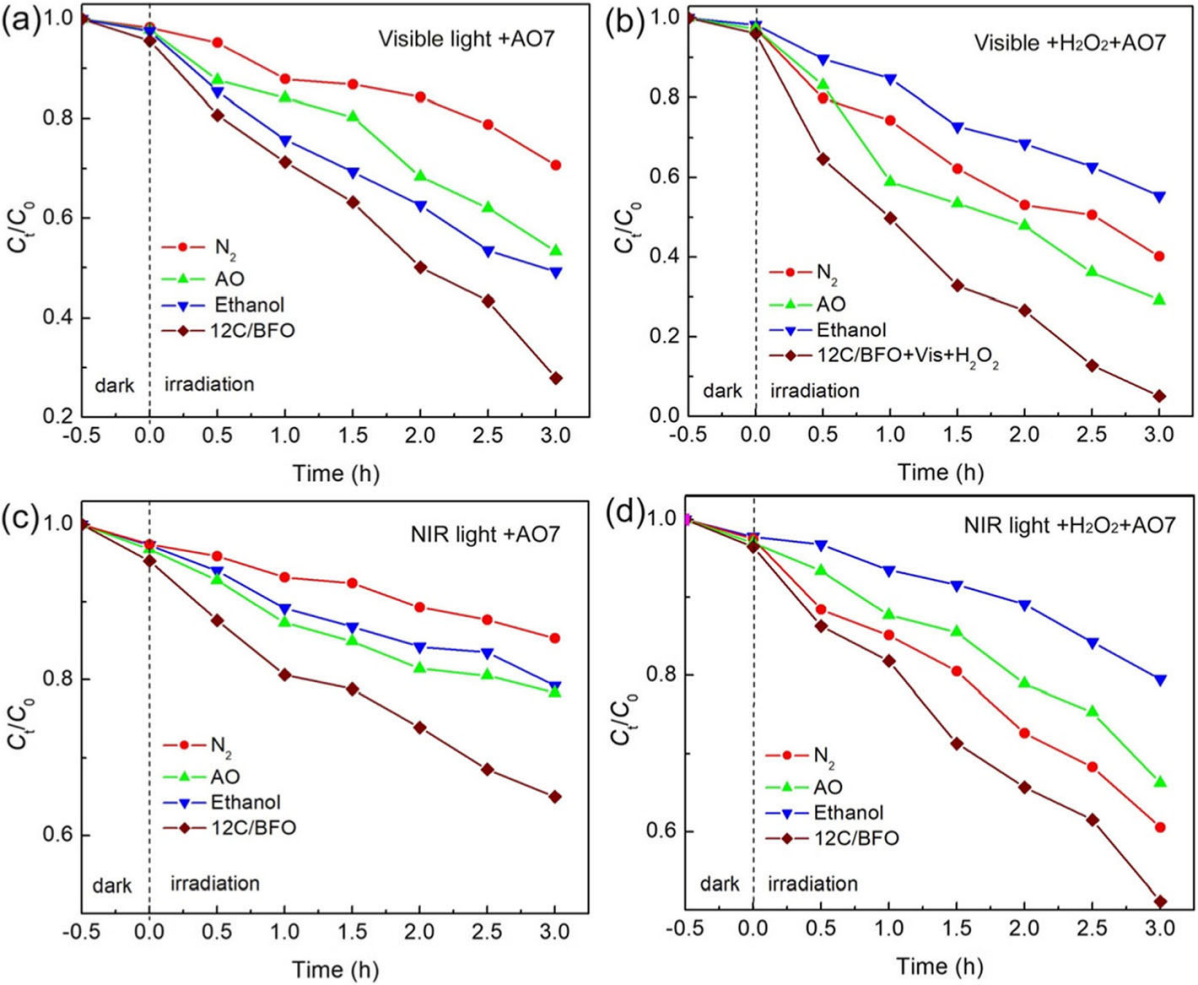
**a** and **b** Effects of ethanol, N_2_ purging, and AO on the photocatalytic and photo-Fenton catalytic degradation of AO7 over 12C/BFO under visible light irradiation, respectively. **c** and **d** Effects of ethanol, N_2_ purging, and AO on the photocatalytic and photo-Fenton catalytic degradation of AO7 over 12C/BFO under NIR light irradiation, respectively

To explore the effect of active species on the catalytic degradation reaction, reactive species trapping experiments were carried out. Figure 10a and b show the photocatalytic and photo-Fenton catalytic degradation of AO7 using the 12C/BFO sample with the addition of quenchers under visible light illumination, respectively. From Fig. 10a, the introduction of ethanol and AO leads to relatively small inhibition on the AO7 degradation. In contrast, the photocatalytic degradation of AO7 is dramatically suppressed with N_2_ purging. This suggests that the ·O_2_^−^ is the primary reactive species, whereas ·OH and h^+^ are the secondary reactive species responsible for the dye degradation. As shown in Fig. 10b, the degradation percentage of AO7 decreases from 96% (without scavengers) separately to ~ 60% (N_2_ purging), ~ 71% (adding AO), and ~ 45% (adding ethanol). This reveals that ·O_2_^−^, h^+^, and ·OH participate in the visible light-driven photo-Fenton catalytic reaction, and ·OH plays a relatively large role in this process. Figure 10c and d present the photocatalytic and photo-Fenton catalytic degradation of AO7 over the 12C/BFO sample in the presence of scavengers with the irradiation of NIR light, respectively. It can be seen that in the both catalytic processes, the dye degradation depends on ·O_2_^−^, h^+^, and ·OH. Particularly, ·O_2_^−^ is demonstrated to be the main active species in the NIR light-driven photocatalytic process, whereas ·OH exhibits a key duty in the NIR light photo-Fenton catalytic reaction.

**Fig. 11:**
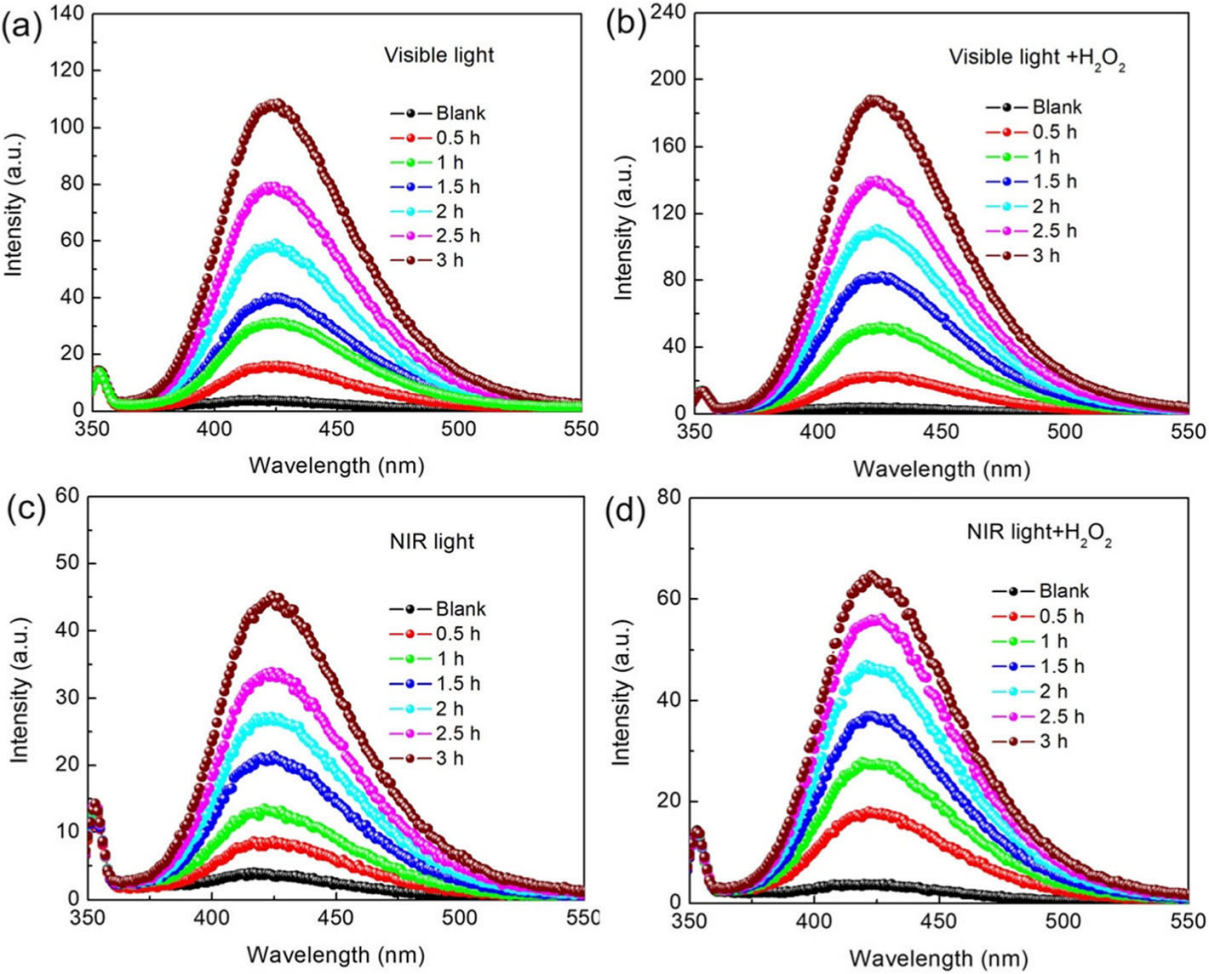
**a** and **b** PL spectra of the TA solution as a function of visible light irradiation time over the 12C/BFO sample in the photocatalytic and photo-Fenton catalytic reactions, respectively. **c** and **d** PL spectra of the TA solution as a function of NIR light irradiation time over the 12C/BFO sample in the photocatalytic and photo-Fenton catalytic reactions, respectively

Figure 11 displays the time-dependent PL spectra of the TPA solution using the 12C/BFO sample as the catalyst in the photocatalytic and photo-Fenton catalytic reaction under visible and NIR light illumination. It is seen that, in all cases of the catalytic processes, the PL emission peak located at ~ 429 nm becomes intense gradually with the increase of the illumination time, indicating the generation of ·OH radicals. Based on the PL signal intensity, it is concluded that more ·OH radicals are generated in the photo-Fenton process than in the photocatalytic process, and the visible light irradiation leads to the increased generation of ·OH radicals when compared with the NIR light irradiation.

### Photogenerated Charges Performance

**Fig. 12:**
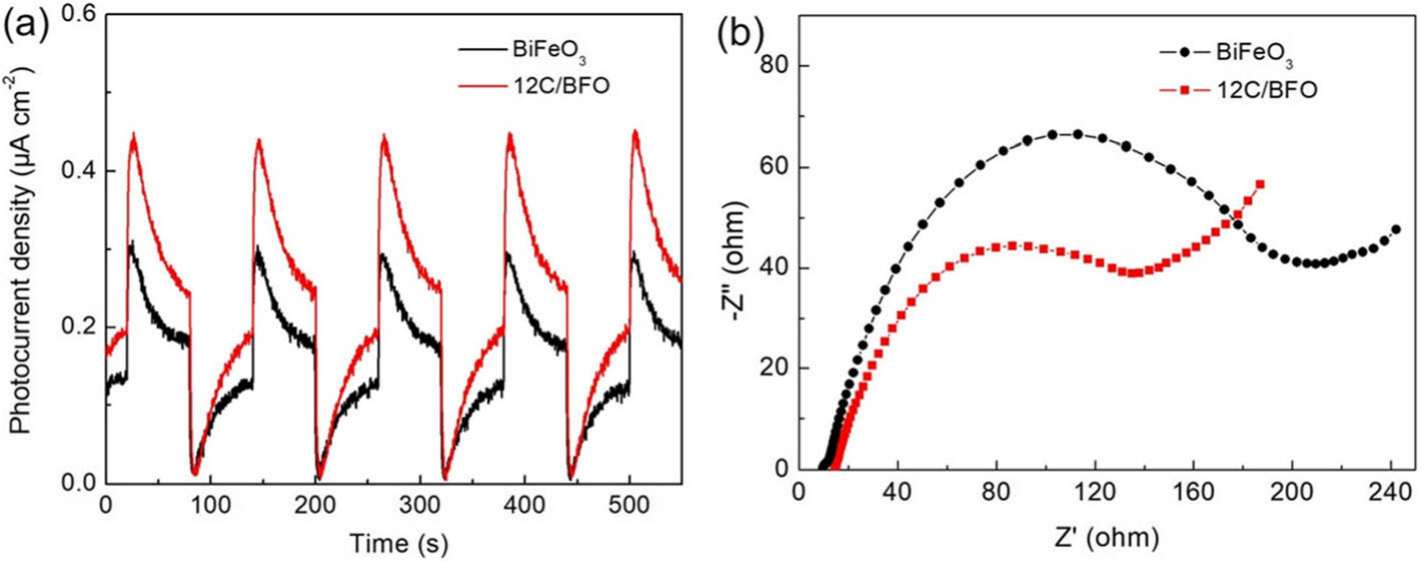
**a** Photocurrent response plots of BiFeO_3_ and 12C/BFO under visible light irradiation. **b** EIS spectra of BiFeO_3_ and 12C/BFO

Photoelectrochemical measurement is very useful for the investigation of the migration and recombination performance of photogenerated charges. The transient photoresponse currents of BiFeO_3_ and 12C/BFO under visible light irradiation with several on/off cycles are shown in Fig. 12a. One can see that the photocurrent density of 12C/BFO is much higher than that of bare BiFeO_3_, indicating the effective separation of photogenerated charges in the CQDs/BiFeO_3_ composite. Figure 12b displays the EIS curves of BiFeO_3_ and 12C/BFO. It is well known that the semicircle in the Nyquist plot at the high-frequency region reflects the interfacial charge-transfer process and a smaller diameter of semicircle means a lower charge-transfer resistance [66]. The 12C/BFO sample exhibits a smaller semicircle diameter compared with bare BiFeO_3_, suggesting that the migration of photogenerated charges can be promoted in the CQD/BiFeO_3_ composites.

### Catalytic Mechanism

**Fig. 13:**
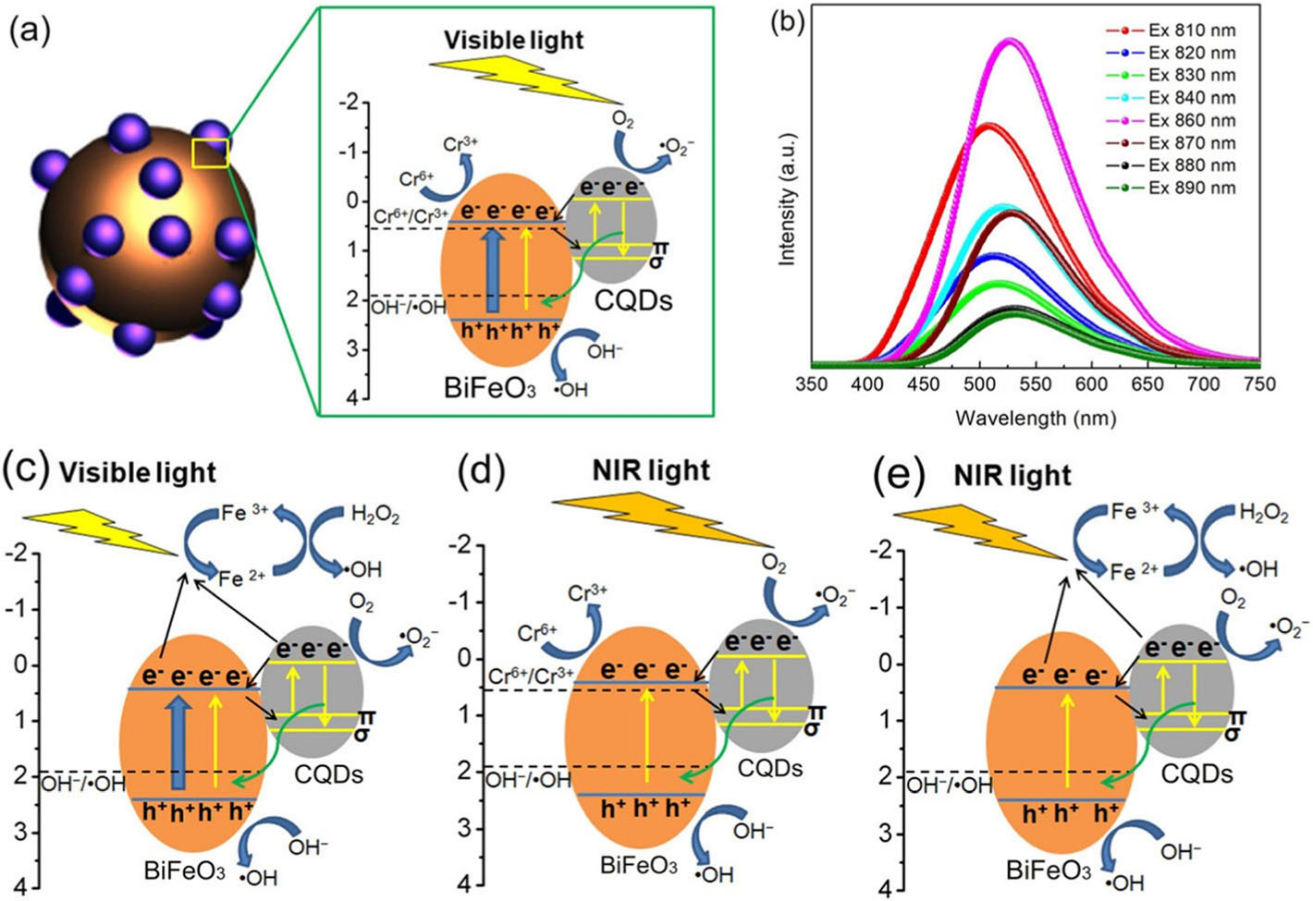
**a** Photocatalytic degradation mechanism of AO7 over the CQDs/BiFeO_3_ composite photocatalysts under visible light irradiation. **b** Up-converted PL spectra of CQDs under different excitation wavelengths. **c** Photo-Fenton catalytic degradation mechanism of AO7 over the CQD/BiFeO_3_ composites under visible light irradiation. **d** NIR light photocatalytic mechanism of the CQDs/BiFeO_3_ composites. **e** NIR light photo-Fenton mechanism of the CQD/BiFeO_3_ composites

A possible visible light-driven photocatalytic mechanism of CQDs/BiFeO_3_ for the dye degradation and Cr(VI) reduction is proposed, as shown in Fig. 13a. When the CQD/BiFeO_3_ composite is irradiated by visible light, the BiFeO_3_ nanoparticles will be excited to generate photogenerated electrons and holes. On the other hand, the electrons in the CQDs can be also excited from their *π* orbital or *σ* orbital to the lowest unoccupied molecular orbital (LUMO) to obtain photoexcited electrons. It has been demonstrated that the excited CQDs can act as excellent electron donors and electron acceptors. Therefore, the photogenerated electrons in the conduction band (CB) of BiFeO_3_ nanoparticles will easily migrate to the *π* orbital or *σ* orbital of CQDs, while the photoexcited electrons of CQDs will transfer to the CB of BiFeO_3_. During the above converse electron migration process, the separation of photogenerated charges in BiFeO_3_ can be promoted, as revealed by photoelectrochemical measurement (see Fig. 12a). Thus, more photogenerated charges are available for participating in the photocatalytic reaction, leading to the improvement of photocatalytic activity.

More importantly, the up-converted PL property of CQDs also plays an important role in the enhancement of photocatalytic activity. Figure 13b presents the up-converted PL spectra of CQDs with the excitation wavelength from 810 to 890 nm, from which one can see that the up-converted emission peaks are centered at shorter wavelengths in the range of 400–680 nm. Because the light absorption edge of the as-prepared BiFeO_3_ nanoparticles is located at ~ 588 nm (see Fig. 4), the up-converted emission light (400–588 nm) of CQDs can be used to excite BiFeO_3_ nanoparticles to produce photogenerated electrons and holes, which provides additional photogenerated charges for the photocatalytic reaction. This also contributes to the enhancement of photocatalytic activity for BiFeO_3_ nanoparticles.

Besides the yield of photogenerated charges, the redox ability of photogenerated charges is considered to be another important factor for understanding the catalytic mechanism of catalysts. In our previous work, the CB and VB potentials of prepared BiFeO_3_ nanoparticles are calculated to be + 0.4 and + 2.47 V vs. NHE, respectively [55]. From a thermodynamic point of view, the generation of ·OH will be smoothly achieved because the VB potential of BiFeO_3_ is more positive than the redox potential of OH^−^/·OH (+ 1.99 V vs. NHE) [67]. Compared with the redox potential of Cr(VI)/Cr(III) (+ 0.51 V vs. NHE) [57], the photogenerated electrons in the CB of BiFeO_3_ is negative enough to reduce Cr(VI) to Cr(III). Another active species ·O_2_^−^ can be obtained from the reaction between the photoexcited electrons of CQDs and O_2_ [68].

Figure 13c presents the visible light-driven photo-Fenton catalytic degradation mechanism of the dye over the CQD/BiFeO_3_ composites. In this case, the photocatalytic and Fenton reactions will simultaneously happen. When H_2_O_2_ is introduced into visible light-driven photocatalytic system, the H_2_O_2_ can react with Fe^2+^ on the surface of BiFeO_3_ to obtain additional ·OH along with the generation of Fe^3+^. Simultaneously, the Fe^3+^ will be reduced to Fe^2+^ by the photogenerated electrons of BiFeO_3_ and CQDs [69]. During this cycle reaction, more ·OH is produced, which is beneficial for the enhancement of catalytic efficiency.

Figure 13d and e display the photocatalytic and photo-Fenton catalytic mechanism of the CQDs/BiFeO_3_ composite under NIR light irradiation. It is known that the BiFeO_3_ do not response to NIR light (> 800 nm). As a result, only CQDs can be excited under NIR light irradiation in the two catalytic processes. The photogenerated charges migration and up-converted excitation of CQDs are similar to those as depicted in Fig. 13a and b. Because the BiFeO_3_ cannot be directly excited by NIR light, NIR light-excited CQD/BiFeO_3_ composite has a relatively lower yield of photogenerated charges compared with the visible light-excited composite. This is why photocatalytic and photo-Fenton catalytic activities of the CQD/BiFeO_3_ composites under NIR light irradiation are weaker than those under visible light irradiation.

## Conclusions

The CQDs were successfully decorated on the surface of BiFeO_3_ nanoparticles through a hydrothermal route to obtain CQD/BiFeO_3_ composites. Under visible and NIR light irradiation, these composites manifest remarkably enhanced photocatalytic degradation of AO7, photocatalytic reduction of Cr(VI), and photo-Fenton catalytic degradation of AO7 compared with bare BiFeO_3_ nanoparticles. They can be reused without obvious decrease of catalytic activities. It is found that the introduction of CQDs leads to the efficient separation of photogenerated charges in the composites. The improved catalytic activities of CQD/BiFeO_3_ composites can be ascribed to the two factors: the excellent up-converted photoluminescence property and photogenerated electron transfer ability of CQDs.

## Data Availability

All data analyzed during this investigation are presented in this article.

## Abbreviations

AO: Ammonium oxalate
AO7: Acid orange 7
CB: Conduction band
CQDs: Carbon quantum dots
Cr(VI): Hexavalent chromium
DF-STEM: Dark-field scanning transmission electron microscope
DPC: Diphenylcarbazide method
DRS: UV-vis diffuse reflectance spectra
*E*_g_: Bandgap energy
EIS: Electrochemical impedance spectroscopy
FTIR: Fourier-transform infrared spectroscopy
h^+^: Photogenerated holes
HRTEM: High-resolution transmission electron microscope
LUMO: Lowest unoccupied molecular orbital
NIR: Near-infrared light
O_2_^−^: Superoxide radical
OH: Hydroxyl radical
PL: Photoluminescence
TA: Terephthalic acid
TAOH: 2-Hydroxyterephthalic acid
TEM: Transmission electron microscope
VB: Valence band
XPS: X-ray photoelectron spectroscopy
XRD: X-ray diffractometer
